# Verbal Working Memory as Emergent from Language Comprehension and Production

**DOI:** 10.3389/fnhum.2020.00068

**Published:** 2020-03-12

**Authors:** Steven C. Schwering, Maryellen C. MacDonald

**Affiliations:** Department of Psychology, University of Wisconsin-Madison, Madison, WI, United States

**Keywords:** working memory, language comprehension, language production, serial order, long-term memory, lexical representations

## Abstract

This article reviews current models of verbal working memory and considers the role of language comprehension and long-term memory in the ability to maintain and order verbal information for short periods of time. While all models of verbal working memory posit some interaction with long-term memory, few have considered the character of these long-term representations or how they might affect performance on verbal working memory tasks. Similarly, few models have considered how comprehension processes and production processes might affect performance in verbal working memory tasks. Modern theories of comprehension emphasize that people learn a vast web of correlated information about the language and the world and must activate that information from long-term memory to cope with the demands of language input. To date, there has been little consideration in theories of verbal working memory for how this rich input from comprehension would affect the nature of temporary memory. There has also been relatively little attention to the degree to which language production processes naturally manage serial order of verbal information. The authors argue for an emergent model of verbal working memory supported by a rich, distributed long-term memory for language. On this view, comprehension processes provide encoding in verbal working memory tasks, and production processes maintenance, serial ordering, and recall. Moreover, the computational capacity to maintain and order information varies with language experience. Implications for theories of working memory, comprehension, and production are considered.

## Introduction

When Ebbinghaus (1885) published his extensive verbal memory experiments and observations, he established a new theoretical approach to cognitive psychology through the formal study of memory. In his quest to isolate the properties of memory, Ebbinghaus observed that immediate recall of verbal material was utterly contaminated by long-term knowledge of the language. He found it impossible to isolate immediate memory when he probed recall of meaningful verbal memoranda such as lines of poetry or narratives, and he established critical methodological practices aimed at stripping away confounding factors. In his attempt to isolate immediate memory, Ebbinghaus developed a collection of nonwords, thousands of consonant-vowel-consonant syllables that could be used to construct lists for immediate recall. The contamination of long-term experience persisted, as certain nonwords exhibited “very important and almost incomprehensible variations as to the ease or difficulty with which they are learned” (p. 23). Moreover, Ebbinghaus noted that even these novel materials could not completely isolate immediate memory from other cognitive processes; visual, acoustic, and articulatory components of verbal perception and action necessarily affected task performance.

Over 130 years of research now contributes to answering the questions posed by Ebbinghaus, and it is useful to ask how his catalyzing observations continue to influence theoretical and methodological approaches to memory research. In this article, we critically analyze Ebbinghaus’s goal of isolating immediate memory as well as his warning that such isolation may be impossible. Following some establishment of terms and definitions and a brief sketch of some current models of immediate memory, we consider several intersecting points, all of which stem from a language-based perspective on the ability to temporarily maintain verbal information. First, we consider the dependence of immediate memory on long-term language knowledge, as Ebbinghaus first observed, and consider the impact of these relationships on modern theories of working memory. These modern accounts recognize some role for long-term memory, but we argue that they have been slow to embrace more modern approaches to the nature of long-term word representations and processing. Instead, we argue that language comprehension and production processes underpin encoding, maintenance, and production of old and new verbal memoranda without the need for separable buffers that are common in some current memory models. A key development in some models of immediate memory is the assumption that memory for words is separate from memory for their orders. In contrast, we consider the many ways in which various word and order representations are intertwined in language comprehension and production research and propose a new emergent account that incorporates these representations in VWM. In closing, we consider the implications of our perspective on theories of language use and on related research areas.

## Working Memory Models and Terminology

There exists a fundamental disagreement about the definition of working memory (e.g., Cowan, 2008; Aben et al., 2012), as evidenced by a wide array of both qualitative descriptions of immediate memory and competing memory models (see Cowan, 2017). We will focus on two general classes of models for how humans can encode verbal material, maintain it for a brief period of time, and produce the memoranda by speaking or writing. Proponents of the two types of models that we discuss, the multi-component models (e.g., Baddeley et al., 1975; Baddeley, 2000) and emergent models (e.g., Cowan, 1993; Postle, 2006), do not always use terms in the same way, and so we begin with some definitions.

Verbal working memory (VWM) is commonly viewed as the temporary maintenance of verbal information (i.e., some aspects of language). Some researchers distinguish VWM as an immediate memory for processing of information (converting speech to meaning, say) from short-term memory (STM), a passive temporary store. However, as Buchsbaum and D’Esposito (2019) have noted, information is always being transformed in some way in the service of goal-directed behavior, and so we will use the term VWM to refer to both storage and processing, except where we specifically refer to theories invoking an STM component. Finally, VWM researchers have increasingly investigated the ability to recall verbal material in the same order it was presented. Thus, we discuss abilities to recall a word or nonword (termed *item memory*) and recall in the correct order in a list (*order memory*).

Multicomponent models, which get their name from the distinct components posited in the working memory system (Baddeley, 1992), draw a sharp distinction between passive storage of information in “buffers” and processing mechanisms such as speech perception and production processes. In this respect, multicomponent models are aligned with classical theories of working memory advanced by Ebbinghaus. In this view, the sole function of STM is to act as a site of storage. Specifically, multicomponent models posit a short-term buffer that maintains a rapidly degrading representation of memoranda (Baddeley et al., 1984). Critically, in this perspective, long-term memory is separate from STM (e.g., Shallice and Warrington, 1970, 1974), but *via* a process called *redintegration* (e.g., Hulme et al., 1997), LTM can provide cues to rebuild STM as it degrades (Lewandowsky and Farrell, 2000). LTM can interact with STM in other ways. With respect to language processing, some researchers claim that verbal STM is a buffer that stores partially processed linguistic representations (e.g., Martin and Romani, 1994; Martin and He, 2004), or is a specific subcomponent of language processing mechanisms dedicated to storage (Shallice and Papagno, 2019). Certain theories propose that the buffer holds copies of or pointers to representations derived from LTM that may require further processing in the future (Norris, 2017). Thus, whereas Ebbinghaus (1885) tried to isolate STM processes within an interacting system, the multi-component models have converted that research goal into an architectural claim: STM is a distinct system with only the most limited, indirect contact with LTM and language processing mechanisms.

Although multicomponent accounts are the dominant perspective in VWM research, there is a long history of caution about this approach. More than 25 years ago, Crowder (1993) predicted a wholesale reassessment of multi-component models of VWM in favor of alternative approaches. He described the notion of a separate, dedicated short-term store (the multicomponent model) as “archaic and, to some of us, even downright quaint” and suggested that “Increasingly, the field is turning instead to a procedural attitude toward memory” (p. 143). Crowder’s predictions were wildly inaccurate in their timeline, as multi-component models of memory remain important and useful theories of VWM now many decades after Crowder predicted their demise. Nevertheless, Crowder correctly predicted the rise of alternative, emergent models of VWM that did away with separate buffers.

Emergent approaches do not generally distinguish between storage and processing mechanisms. Some earlier variants were called procedural models, defining VWM as a secondary product of procedures in support of other cognitive processes (Craik and Lockhart, 1972; Kolers and Roediger, 1984; Crowder, 1993; Jones et al., 2004). Early theorizing by Saffran and Martin (1997) explored relationships between aphasic patients’ VWM in the context of their language production abilities, informed by Dell (1986) spreading activation model of language production (Martin et al., 1996; Saffran and Martin, 1997). We advocate this “rich emergent” approach here, where VWM is the activated portion of linguistic LTM (Cowan, 1993; Postle, 2006; Acheson and MacDonald, 2009a,b; Hasson et al., 2015; MacDonald, 2016; Buchsbaum and D’Esposito, 2019). This approach emphasizes VWM as a complex of skills, honed by past language comprehension and production experience. In this view, knowledge of word meanings and other forms of linguistic knowledge shape performance in VWM tasks. Performance on VWM tasks co-opts language LTM, by which we mean any parts of LTM involved in language tasks, including knowledge of events, word meanings, word order, phonological form, and other information (MacDonald and Christiansen, 2002; Acheson and MacDonald, 2009b; MacDonald, 2016). LTM itself is characterized as a set of processing mechanisms employed to achieve goal-directed behavior rather than store a static set of memoranda chunked or compressed from prior experience (Postle, 2006; Buchsbaum and D’Esposito, 2019). In the case of WM for linguistic memoranda, we have proposed that the language production architecture is co-opted to maintain and order the memoranda, obviating the need for a separate memory buffer (Acheson and MacDonald, 2009b; MacDonald, 2016). Whereas, in the multicomponent model, effects of prior language knowledge in LTM have been attributed to secondary mechanisms (e.g., Hulme et al., 1997; Lewandowsky and Farrell, 2000), we see these LTM effects arising naturally from language production and comprehension processes. For example, language production is well known to favor serial orders that have been used frequently or recently (Bock, 1986a) and to group related words together in an utterance (Solomon and Pearlmutter, 2004). These biases in production may underlie the effects of semantic grouping and similarity to natural language that has been observed in recall tasks (Miller and Selfridge, 1950; Jones and Farrell, 2018). Thus, we view temporary maintenance and ordering as the job of action systems, which must construct an action plan and maintain it before it can be executed, so that the action plan is the “memory of what is to come” (Rosenbaum et al., 2007, p. 528). For language, the action planning system is language production, and the utterance plan is the memory of both what is to be produced and the order in which it will be produced at several levels, including words, phonemes, and articulatory gestures (Martin et al., 1996; Acheson and MacDonald, 2009b; MacDonald, 2016). In this view, VWM is simply the skill of maintaining and ordering linguistic material, and that skill, as with all subcomponents of language production and comprehension, emerges from actions of the language systems and varies with experience (MacDonald and Christiansen, 2002; MacDonald, 2016).

In contrast to the “rich emergent” account described above, some “limited emergent” accounts posit a more restricted interaction with language processes, with different systems working in parallel to support memory for items and their orders (Majerus, 2013, 2019). On this view, item memory engages ventral language pathways that process semantics, with dorsal pathways supporting order within the item (i.e., phonemes). In contrast, order memory for sequences of words themselves engages frontal-parietal networks and networks closely associated with attentional mechanisms. The item/order memory distinction has been supported by findings that word characteristics, like frequency of use (Poirier and Saint-Aubin, 1996; Saint-Aubin and Poirier, 1999) and semantics (Majerus and D’Argembeau, 2011), largely affect memory for items but not memory for order. Furthermore, memory for items and order appear to engage distinct neural populations, as indicated by neuroimaging results (Majerus et al., 2006, 2008; Guidali et al., 2019) and aphasic patient data (e.g., Majerus et al., 2007, 2015).

The separate item/order memory of more limited emergent accounts is consistent with a multicomponent approach, namely that LTM is able to support STM only in cases where the items and order conform to prior experience. Multicomponent models are particularly emphatic about this point, arguing that this is a critical reason an STM buffer must exist distinct from LTM (e.g., Norris, 2017). Some emergent accounts also recognize that there are limitations to LTM. For example, Majerus (2013) suggests that “the representations of the language system are able to support familiar item and order information, but not unfamiliar order information” (p. 4). This distinction between familiar and unfamiliar orders is problematic because it presumes a dichotomy between the novel and familiar when similarity to prior experience is actually continuous. We consider this point further in the section entitled “Problems with Limited Emergence.”

In the next sections, we contrast our rich emergent account against a variety of alternative multi-component and more limited emergent memory models. Specifically, we describe current research on the nature of LTM language representations and the language comprehension and production processes that interact with LTM. Because all accounts of VWM must refer in some way to LTM, we argue that this characterization of language knowledge informs all theories of encoding, maintaining, and ordering verbal information.

## Word Representations in VWM and Language Research: No Word Is an Island

Since the time of Ebbinghaus, most VWM models have assumed discrete representations or “items” in memory. Often, verbal memory is conceptualized by the unit of the word or word-like collections of phonemes (nonwords). For example, there are a multitude of studies investigating immediate or delayed word recall that document word accuracy across list position (e.g., Murdock, 1962; Watkins and Watkins, 1977), word omissions (e.g., Roodenrys et al., 2002), word intrusions (e.g., Coltheart, 1993), and so on. Furthermore, measurement of VWM capacity is often indexed by list span, or the average number of words recalled from lists (e.g., Daneman and Carpenter, 1980; Hulme and Tordoff, 1989). In part, such descriptions are a convenient shorthand for bits of information (Miller, 1956), but they also reflect certain assumptions about the isolability of memory representations. One common assumption is that word memory is supported by fully separable phonological and semantic codes (Martin, 1987; Martin et al., 1999; Howard and Nickels, 2005). Another is that order memory is separable from the memory for the word, itself; this view is further compounded by viewing the words in lists as separate from each other, especially in the case of novel word orders (Majerus, 2013, 2019).

Considering that all major memory models posit some kinds of ties with language representations, it bears asking how a compartmentalized view of item and order representations, and a compartmentalized view of item components (e.g., phonology, semantics, grammatical role), accords with language research. In this section, we describe developments in both comprehension and production research that is completely antithetical to the isolated representations prevalent in much memory research. This work shows that different levels of language representation used in production and comprehension, what we refer to as language LTM, influence each other and are integrated. We suggest that this integration, and the statistical regularities between classically defined and supposedly dissociable representations that are critical for language research, have significant consequences for how verbal information is maintained. In other words, we argue that the nature of linguistic LTM representations, as revealed in research on language comprehension and production, is highly relevant to theories of VWM.

### Integrated Representations in Language Processing

Researchers’ views about the nature of word representations and their use in comprehension and production have undergone enormous change in the last several decades. Initially, researchers believed that comprehension processes were modular, such that dedicated components worked independently to interpret language input (e.g., Seidenberg et al., 1982; Frazier, 1987; see also Almeida and Gleitman, 2018 for more historical context and current views of modularity). Similarly, models of production were highly staged, with minimal interaction between different language representations (e.g., Levelt et al., 1999). Theories of word representation pointed to a lexicon with distinct levels (phonological, syntactic, semantic, e.g., Allport and Funnell, 1981). Importantly, these models assumed that, regardless of the nature of LTM, language processes could selectively extract and operate over subcomponents of linguistic knowledge, such as processing phonology or syntax without meaning, with some later integration stage (Forster, 1985; Frazier, 1987). While this work did not often invoke VWM, the notions of separable language components and isolated processing systems are compatible with the orientation of multi-component models.

More recent theories of language comprehension are far less aligned with these compartmentalized approaches. Instead, they have emphasized extensive interaction between different kinds of language representations. This is most clearly demonstrated behaviorally in instances where certain information cannot be “turned off,” even when it is beneficial to do so (e.g., Stroop, 1935). For example, Seidenberg and Tanenhaus (1979) demonstrated that the orthographic form of a word interfered with judgments of phonological form, meaning that one form of information in LTM (orthographic information) interfered with another form of information in LTM (phonological form). While early neuropsychological studies suggested that the subcomponents of language knowledge were represented with discrete neural codes (Dapretto and Bookheimer, 1999), more recent analyses support integrated representations. For example, Siegelman et al. (2019) argue against previous evidence for divisions between syntactic and semantic representations during sentence comprehension. Similalry, Dikker et al. (2010) found that phonological/orthographic information contributes to syntactic analyses within 100 ms, even before a word has been recognized because the phonological form is correlated with, and therefore provides information about, the likely grammatical category (noun, verb, etc.) of the to-be-recognized word. Together, this work and others (e.g., Pereira et al., 2018) suggest that word comprehension and LTM representations are much more interconnected than was previously recognized.

This article is not the place for a full specification of how representations are integrated, nor for the natural ongoing debates concerning how to characterize linguistic knowledge, but it is worth noting why a number of researchers now assume extensive interaction and integration among what has been traditionally described as distinct levels of linguistic information. In more integrated accounts, multiple sources of information interact in perception and comprehension because interactions are beneficial, essential really, to comprehend and produce language in real-time. Language contains strong correlations between different levels of representation, between language and the world, and between information earlier and later in a linguistic signal to be interpreted. People are voracious statistical learners, and they leverage their LTM of the statistical regularities between different kinds of information to comprehend and produce language efficiently and accurately (Seidenberg and MacDonald, 2018). Indeed, the combination of several partially informative information sources (phonology and semantics, for example) is now seen as central to accounting for the speed with which comprehenders interpret incoming language input despite the massive ambiguity known to pervade language; an individual source of information only weakly constrains interpretation alone but is highly effective in combination with other constraints (Seidenberg, 1997; MacDonald and Seidenberg, 2006; Graves et al., 2010; Joanisse and McClelland, 2015). Each language comprehension experience is a source of learning (Chang et al., 2000), and a consequence of learning all this combinatorial information is that any single source of information, including words, cannot be atomic or isolated (Willits et al., 2015). Instead, words and other classically defined levels of representation are highly intertwined, because learning (and therefore LTM) must capture a complex web of statistical structure to maximize performance during language comprehension and production. Word representations can be modeled as attractors in networks comprising various types of information (phonological, semantic, etc., Hinton and Shallice, 1991), and some linguists and psycholinguists now consider discrete notions such as *word* and *phoneme* to be convenient fictions, highly useful for researchers’ discussions but having more to do with people’s conscious intuitions than with the way that language is actually represented and processed in the brain (Bybee and McClelland, 2005; Baayen et al., 2016; Ramscar and Port, 2016).

### Separated Representations in Memory-Models

These highly interactive approaches have not yet penetrated much of the theorizing in most current multi-component and emergent models of VWM, which continue to emphasize individual “items” of memory. Multicomponent models posit specialized, separate buffers, such as the phonological loop (Baddeley and Hitch, 1974), which encode a single type of information. Initially, patient lesion data seemed to provide further support to modular memory and language approaches, as in patients who exhibited impaired memory abilities with spared language abilities (often called “STM patients,” e.g., Warrington and Shallice, 1969) and in cases reporting double dissociations of phonological and semantic information in memory and language tasks, leading to a separation between phonology and semantics in multicomponent models (Martin and Romani, 1994; Martin et al., 1994). This dissociation between representations extends into memory for items and their order. Certain aphasic patients demonstrate apparently isolable item or order memory impairments (Attout et al., 2012; Majerus et al., 2015), and this behavioral pattern is accompanied by neuroimaging evidence suggesting item and order memory are supported by distinct neural populations (Kalm and Norris, 2014; Attout et al., 2019).

A strict notion of “item” in memory becomes more complicated when considering the qualities of statistical information in linguistic LTM. For example, phonotactic long-term knowledge influences recall of novel words. Non-words consistent with the transitional probabilities of phonemes (or acoustic properties or articulatory gestures) in natural language are recalled better than non-words inconsistent with these patterns (Gathercole et al., 1999; Thorn and Frankish, 2005). Researchers have likewise extended these findings to suggest that both lexical and sublexical properties affect recall of non-words (Roodenrys et al., 2002; Majerus et al., 2004). Tanida et al. (2019) further demonstrated an effect of forward and backward bimora transition probabilities on ordered recall. Together, these results suggest that memory of one phoneme or acoustic pattern influences memory of others *via* LTM of the phonological statistical structure of language. These “neighborhoods” of patterns in LTM can be quite subtle, as evidenced by the improved recall for nonwords with regular pitch accent compared to irregular pitch accent, an effect moderated by phonotactic frequency (Tanida et al., 2015; see also Yuzawa and Saito, 2006). Not only do these studies suggest that LTM is relevant for VWM, but they suggest multiple grain sizes of phonological information interact to inform performance in memory tasks.

Beyond phonological information, language users also track and leverage complex statistical regularities between different types of linguistic representations, such as between phonology and semantics. Our claim is not that phonology and semantics are completely merged (they are clearly not), but rather that they are intertwined to a degree that affects language use and VWM performance. Such regularities are not always obvious. Indeed, with some exceptions (Farmer et al., 2006; Schmidtke et al., 2014; Christiansen and Monaghan, 2016), the mapping between phonology and semantics seems largely arbitrary. If phonology and semantics were completely distinct, then each representation could be stored in a separable buffer, consistent with multicomponent accounts. However, claims for a strict semantic-phonological divide break down when considering morphologically complex words, such as *painter, ideas, friendship*, and *working*. These words contain morphemes (-*er, -s, -ship, -ing*) for which the mapping from phonology to semantics is not arbitrary. The same mapping occurs repeatedly through the language (e.g., *worker, baker, seeker*, etc.), and words sharing these affixes form semantic-phonological neighborhoods that shape language LTM and behavior (Rueckl et al., 1997; Seidenberg and Gonnerman, 2000). These relationships also encode grammatical form (e.g., -*er* is associated with nouns, -*ing* with verbs). It might be tempting to consider morphologically complex words as marginal and not part of more “typical” language, but morphologically complex words are common in English and their phonological-semantic-grammatical regularities have been shown to affect word learning in infants (Willits et al., 2014). In adults, regularities between phonological, orthographic, semantic, and grammatical knowledge drive very early stages of language comprehension, even before conscious word recognition (Dikker et al., 2010). Even so, recent reviews suggest there is a “notorious lack of consensus” (p. 37) in the imaging literature about the brain representations of phonological, semantic, and morphological relationships among more complex words (Leminen et al., 2019). As such, it is clear that many representations simultaneously impact language comprehension and production, and it is unclear how any single representation could be extricated from this web of processing.

Given these regularities in language use, it is not surprising that morphophonological regularities also impact VWM. For example, the use of morphophonological cues has been well-studied in children’s nonword repetition. Nonwords with morphophonological cues are recalled better than nonwords without such cues, and children with language impairments may be less sensitive to this effect (Archibald and Gathercole, 2006; Casalini et al., 2007; Estes et al., 2007). Thus, experience with language, specifically the regular co-occurrences between phonology and semantics in morphologically complex words, affects VWM for nonwords (though see Szewczyk et al., 2018). These results have largely been examined with children completing single word repetition tasks. It would be worthwhile to extend this work to other tasks and populations. Incorporating regularities between phonology and semantics in stimuli (e.g., *via* the use of affixes) could alter the apparent separability of phonology and semantics, as has been suggested by many memory and language studies (e.g., Martin et al., 1994).

The “primary systems” approach to memory and language use begins to incorporate some current insights about language representations and argues for phonology and semantics as separable yet interacting representations (Ueno et al., 2014; Savill et al., 2019). Broadly, this approach supports emergent memory accounts, suggesting that the effects of semantics and phonology on word and non-word recall reflect a balance of processing. For example, when phonological support is weak, semantic support affects recall to a larger degree compared to when phonological support is strong (Savill et al., 2019). In such accounts, the interactions between phonology and semantics emerge from processing in a quasi-regular domain, resulting in integrated representations. Ueno et al. (2014) demonstrated that words with low imageability are recalled worse than words with high imageability (i.e., the effect of semantics), and this effect is exacerbated in words with an atypical pitch accent (i.e., effect of phonotactics). In line with the primary systems account, this suggests that the effect of phonotactics on recall depends in part on semantics. Interestingly, the researchers developed a neurobiologically constrained connectionist model of word comprehension, repetition, and production, demonstrating that phonological (ventral) and semantic (dorsal) language pathways are differentially engaged when processing typical and atypical phonotactic patterns. As a result, the semantic pathway was more engaged in processing atypical phonotactic patterns. Such research suggests that subtle phonological information may infiltrate a putative semantic pathway (see also Jefferies et al., 2005).

The tracking of complex statistical patterns in support of language comprehension, production, and memory is not limited to within-word representations like phonology and semantics; statistical regularities also support the representation of word order. This point gets to the heart of the item vs. order distinction in VWM theorizing. Memory researchers readily agree that sentences are recalled better than scrambled lists of words (Brener, 1940), and this effect scales with list approximation to natural language sequence statistics (Miller and Selfridge, 1950). These effects are typically attributed to semantic coherence or episodic pattern recognition (Baddeley et al., 2009; Allen et al., 2018). However, episodic memory is not sufficient to explain the full range of results. Memory is similarly facilitated for lists of non-words that approximate natural language syntax (Epstein, 1961, 1962). Thus, meaning does not seem to be necessary for the effect. Jones and Farrell (2018) further demonstrated that people are more likely to recall sentence-like lists in an order consistent with syntactic knowledge and that errors are more likely to conform to prior syntactic knowledge than expected by chance (for corpus analyses tying language experience to memory performance, see Perham et al., 2009; Jones et al., 2020). In each case, inter-item information affected memory for order *via* long-term knowledge of language syntax, suggesting that memory for items and their order interact to support each other. For example, experience using English builds an LTM of the word *pull*. The LTM of *pull* not only encodes meaning and sound but also co-occurrence tendencies; *pull* is often is flanked by words denoting animate entities and objects involved in a pulling event (as in *The girl pulled the cart*). We are emphatically not claiming that linguistic knowledge is limited to co-occurrence, merely that such knowledge includes linear relationships and that what might be viewed as multi-word frequency knowledge shapes both language use (Seidenberg and MacDonald, 2018) and memory (Arnon and Snider, 2010). While strict chaining accounts of ordering have generally fallen out of favor in memory research (e.g., Hurlstone et al., 2014), these studies suggest that inter-item associations are not only encoded and leveraged for performance in memory tasks (for discussion, see also Fischer-Baum and McCloskey, 2015) but reinforced by LTM. Such effects are likely amplified by the presence of multi-morphemic words (such as *pulled*), because, as noted above, morphemes such as -*ed* also contain grammatical information and provide cues to inter-word relationships (see Epstein, 1961, 1962). Thus, it is unclear to what extent item knowledge can be separated from order knowledge if the source of the order benefit is derived from the information associated with the individual words.

### The Role of Language Processes in Performing VWM Tasks

If performing a VWM task is dependent on language processes, such as comprehension for encoding (MacDonald and Christiansen, 2002), lexical production for item memory (Page et al., 2007), or sentence production skills for item ordering (Acheson and MacDonald, 2009b; MacDonald, 2016), then theories of VWM must consider how theories of language comprehension and production constrain memory performance. Here, we describe some current models of language comprehension and production with a specific eye toward describing statistical regularities in language and the integrated representations in LTM that capture those regularities. Of course, these models were not explicitly designed to model performance in VWM tasks. There is an essential tension between the complexity of LTM representations and modeling: the more complex and intertwined the representations are thought to be, the more difficult it is to capture this complexity in a computational model. Few explicit emergent models of VWM exist, as some researchers have noted (Norris, 2017), though many models adopt principles consistent with the emergent approach (e.g., Botvinick and Plaut, 2006). However, from the language emergent perspective, theories of language comprehension and production should serve as a useful analog, continuing the role models of language use have played in shaping memory research (e.g., Martin et al., 1994).

In this view, language comprehension and production processes underlie the encoding and retrieval mechanisms posited in memory accounts, respectively. Language comprehension processes extract meaning from input by mapping an input signal to a semantic representation of the entities and events being referred to (MacDonald and Hsiao, 2018). Often, comprehension processes involve partial predictions of upcoming input (Federmeier, 2007; Altmann and Mirković, 2009; Kuperberg and Jaeger, 2016), which means that comprehension processes routinely involve not only semantic integration of words that have been encountered but also generation of serial order expectations among representations of words that are likely upcoming in the input. Similarly, the interpretation of some language input can depend on the material that comes later (Connine and Clifton, 1987; MacDonald, 1994). There are many language comprehension models that depend on integrated representations, variously capturing word segmentation (Christiansen et al., 1998), utterance interpretation without a separate word segmentation stage (Baayen et al., 2016), the learning of phonological forms (Plaut and Kello, 1999), word reading and its relationship to phonology (Seidenberg and McClelland, 1989; Plaut et al., 1996), the learning of grammatical knowledge (Allen and Seidenberg, 1999), behavior in the visual world paradigm (Mayberry et al., 2009), disorders of comprehension in individuals with developmental language disorder (also called specific language impairment, Joanisse and Seidenberg, 2003), and more. In turn, language production models attempt to generate a well-formed utterance from a message representation, either externally motivated in the case of a repetition task or internally generated in the case of self-generated production. Several interactive models exist, capturing lexical selection (i.e., retrieving words from LTM, Dell et al., 1997) and phrase (Dell et al., 1997) or sentence production (Chang et al., 2006; Dell and Chang, 2014). The Lichtheim-2 model implements an account of single-word comprehension and repetition as well as the degradation of those processes in aphasia (Ueno et al., 2011). All of these models share several core features that tie them to the emergent account. In each, learning algorithms, such as backpropagation, encode statistical knowledge in the connection weights updated through experience, forming the model’s LTM. Each of these models also develops a VWM through learning; for example the TRACE model of speech perception (McClelland and Elman, 1986) got its name from the claim that the STM trace of the model emerged from the interacting layers of the network. No separable STM buffers divorced from LTM are employed in any of the above models.

Critically, integrated representations are a core part of these language models, most commonly instantiated as distributed representations in a network. Distributed representations as their name implies, spread a representation over the entire network *via* connection weights between layers. Integrated representations exhibit at least two key ties to distributed representations in connectionist language models. First, integrated representations emerge in processing *via* bidirectional spreading activation between layers, a feature evident in models of human comprehension and production (e.g., Dell, 1986; Seidenberg and McClelland, 1989). Second, the integrated representations blend processed information across the network such that phonological, semantic, lexical, and grammatical information cannot be strictly separated from other types of information (e.g., McClelland et al., 2010). Of course, we are not claiming that language models do not develop certain specializations for phonological, semantic, lexical, grammatical, and other types of information. Instead, specialization is a matter of degree, where complete modularity and complete overlap are less likely than an intermediate state (McClelland et al., 2010). For example, in some models, impairments of a discrete representation (e.g., phonology) disrupt the use of other representations (e.g., semantics), *via* layers that allow interaction between those representations (e.g., Monaghan and Woollams, 2017). Such models are most consistent with primary systems accounts (e.g., Ueno et al., 2014; Savill et al., 2019). In other models, the integrated representations are not as explicit. For example, simple recurrent networks of comprehension and production, allow information to be processed through time. Such networks cross item and order information *via* recurrent connections (Elman, 1991; Joanisse and Seidenberg, 2003; Botvinick and Plaut, 2006), and there is no clear way in which item and order information can be separated.

Distributed representations as they are captured in connectionist models are not the only way to characterize integrated representations. We have focused on variations in distributed connectionist approaches as examples that most clearly embrace the interconnected representations that should affect theorizing about VWM, but other computational approaches could also incorporate integrated representations in processing (e.g., Frank and Goodman, 2014). Furthermore, localist representations, like the one implemented in Dell et al. (1997), also have interaction among different types of information and have proven incredibly useful in describing mechanisms by which LTM engages with VWM.

### Potential Research Directions and Predictions for a Language-Emergent VWM

There are several predictions for VWM research that stem from the language emergent view, the first of which emphasizes the role of language production processes in the serial ordering of the items in a memory list. Previous research has argued that production processes are engaged in maintenance and recall of verbal material, specifically that the utterance plan that maintains the to-be-uttered words in order also serves the maintenance and ordering functions during VWM tasks (Acheson and MacDonald, 2009b; MacDonald, 2016). As MacDonald (2016) discussed, this claim is much more controversial for some kinds of VWM tasks and performance than others. For example, Page et al. (2007) posited a limited role for language production processes in ordering at the item level. They argued that parallels between word production processes and word recall in VWM tasks pointed to individual, word-level utterance plans playing a role in phonological maintenance in VWM, but ordering the words themselves (order memory) must be the purview of a dedicated short-term store. Lombardi and Potter (1992) and Potter and Lombardi (1998) hypothesized a different role for language processing: in VWM tasks involving whole sentence repetition, the comprehension system interprets the meaning of the sentence and the production system regenerates it from that meaning. The model we advocate incorporates the language system for remembering individual words, whole sentences, as well as all cases in-between, including ordering of word sequences that are less than full, coherent sentences. As there are very few tests of these ideas in the existing literature, our discussion addresses the kinds of word-ordering phenomena in language production that may be relevant to performance in VWM tasks.

An essential task in language production is the creation of serial order over many levels, including messages, words, sub-lexical forms such as phonemes, and articulatory gestures that enable overt language (Dell et al., 1997). Acheson and MacDonald (2009b) extensively reviewed how the interactivity of phonological information with other information predicted serial order phenomena through the lens of language production research. They concluded that “…one key insight about the serial ordering of verbal information in language production is that serial ordering results from interactions across multiple levels of representation over time, that is to say, as a result of recurrent connectivity” (p. 54). For example, word ordering in language production is more likely to go awry when words share features, including both grammatical features (e.g., noun) and phonological features (Dell and Reich, 1981), meaning that phonological and lexico-grammatical information are together affecting serial ordering processes. Given Acheson and MacDonald’s review, we do not focus on phonological interactions with word order here, but it is worth noting a few more recent phenomena relevant to their claims. A number of studies have investigated semantic-phonological interactions termed semantic binding, the finding that lexico-semantic knowledge affects the nature of phonological representations in VWM and other tasks (e.g., Patterson et al., 1994; Hoffman et al., 2009; Savill et al., 2017). Relatedly, Acheson and colleagues conducted several studies suggesting that phonological and semantic information jointly affect serial order in VWM tasks in a way that would be expected from how information interacts in comprehension and production (Acheson et al., 2010, 2011b; see also Poirier et al., 2014). Similarly, Macken et al. (2014) investigated the memory implications for prosody, the intonation patterns that span whole phrases and sentences in everyday language use, in VWM tasks. Like syntactic and discourse relations, prosody is another multi-word phenomenon that does not fit neatly into the item/order distinctions in memory tasks. Macken et al. (2014) found that prosodic phrasing does affect recall, which argues against individual word units in memory.

Far less research concerns the nature of sentence-level language planning and serial ordering in VWM tasks. We mention three findings from language production research that seem particularly relevant to claims about the role of language production in VWM. All three point to the essential non-independence of words and word orders in utterance planning. First, a central tenet across essentially all approaches to language production is that lexico-semantic characteristics of individual words strongly affect their order in a sentence (Bock, 1987; Levelt, 1993). An example is that animate entities like *woman* tend to appear earlier in utterances than inanimate words like *book*. This effect is thought to reflect a more general phenomenon linked to LTM retrieval, in which early-retrieved words enter the utterance plan first and end up in earlier positions in the utterance (Bock, 1987). Semantic features such as animacy affect retrieval and, consequently, serial position in the sentence (Bock, 1987; MacDonald, 2013). Second, the word orders that people produce tend to be ones that have been recently produced (Weiner and Labov, 1983; Bock, 1986b), but the strength of this effect is modulated by the particular words in the sentence: repeated words lead to more repeated word orders (for review, see Pickering and Ferreira, 2008). Again, words and their orders are interdependent. Third, word orders and the presence/absence of optional words in sentences vary with semantic relationships between words, where semantic similarity between two words yields more word omissions and different word orders than in the absence of semantic similarity across words (Gennari et al., 2012; Hsiao et al., 2014; Montag et al., 2017). Thus, whereas the first two examples illustrated interactions between properties of a particular word and word order of an entire utterance, this example shows that semantic relationships between two words also affect word order. All of these examples of word and word order interdependence are broadly compatible with models of language production that represent production as activation of learned weights in a connectionist architecture; these representations arguably cross item and order memory (Chang et al., 2006; Dell and Chang, 2014; McCauley and Christiansen, 2014). In this view, language production models could serve as highly informative models of serial recall, especially when the models engage in sentence repetition (see Ueno et al., 2011 for word repetition and Fischer-Baum, 2018 for other potential commonalities in serial order representations). We see this approach as inconsistent with the currently dominant views of VWM, that memory for items (the words) and memory for their serial order are unrelated, accomplished by independent mechanisms (Henson et al., 2003; Majerus, 2009; Guidali et al., 2019).

These results and approaches offer several avenues for investigations of the relationship between serial ordering of words in language production and VWM tasks. For example, it is worth further consideration of the item-order distinction in some theories of VWM, particularly those that posit a role for LTM and language production for item memory but a special-purpose system for ordering the items (Page et al., 2007; Majerus, 2009). From the point of language production, serial order is crucial both across items (i.e., word order) and within items (syllable, phoneme, articulatory gesture orders). It is curious that within-word serial order demands are considered “item memory” rather than another example of ordering memory. For current purposes, a key difference between the two types of serial order would seem to be their regularity, in that phonological order is much more rigid than syntactic order. For example, the phonemes and articulatory gestures must be in a particular order to produce a given word, and the semantic identity of the word “binds” the sub-lexical representations and their order together—the semantic binding hypothesis (Patterson et al., 1994). Dell and Chang (2014) posit a similar kind of binding from message-level semantics to the serial orders of words, but this binding is weaker and more variable than in the word-phoneme case; there are statistical regularities between types of messages and sentence forms, but messages can usually also be conveyed with alternative word orders (MacDonald, 2016). In other words, the item-order distinction is really one of two different kinds of serial ordering demands and LTM, and the one called “item memory” (which includes the ordering of phonological codes) is much stronger and more regular than the one called “order memory.” On that view, it should be possible to manipulate these contingencies in simulations or experimentally, perhaps with artificial languages in which “word” order and “phoneme” order vary in their rigidity. If, after learning the artificial language, participants had to perform a memory task, we predict that performance at both levels should respond to the regularities of past experience and thus strength of LTM constraints, in contrast to accounts positing a rigid item/order distinction (see also Acheson and MacDonald, 2009a for discussion of “item” vs. phoneme errors and Botvinick and Bylsma, 2005 for recall in artificial languages).

Another interesting domain is performance in Hebb repetition tasks, in which participants repeatedly encounter certain serial orders across lists (Page et al., 2013; Guerrette et al., 2018). Performance in these tasks should at least initially be moderated by statistical regularities in the broader language (that is, in LTM, *via* prior experience with language), where certain words occur in certain serial orders more frequently than others. For example, we might expect that words referring to animate entities (*child, teacher*) would yield different serial order behavior than inanimate words (*book, table*) in ordered recall, because people’s broader experience ordering different types of words in their history of language production would affect how rapidly repeated patterns are learned. More generally, we expect serial ordering behavior to reflect both long-term language use and also rapid adaptation to more recent ordering contexts, a phenomenon that is robust in both language comprehension (Fine et al., 2013) and production (Bock, 1986a). Whereas Hebb repetition effects have been described in terms of repetition of specific tokens, syntactic priming effects in language processing carry across multi-word grammatical and semantic relations. If there are interactive representations between word and grammatical roles, then classic Hebb repetition effects should carry across these abstract relational categories and be moderated by fit with the category. Indeed, some studies have begun to examine these effects in sentence repetition (Allen et al., 2018; Jones and Farrell, 2018) and in recall of lists with grammatical dependencies (Perham et al., 2009) by considering how lists consistent with grammatical knowledge are recalled better than lists inconsistent with these patterns. The emergent account described here would further predict that the effect of grammatical knowledge would be moderated by semantic information of words, such as animacy, and morphophonological cues, reflecting interrelationships in LTM. For example, recall of animate nouns should be greater than recall of inanimate nouns in the context of word lists that encourage a noun to be interpreted as an agent, because animate nouns are commonly agents of actions and inanimate nouns are not. Furthermore, this account would suggest rapid adaptation to novel orders would affect memory in a manner consistent with models of language production that learn over experience.

### Challenges for the Multicomponent Approach

Rather than viewing memory representations as graded, integrated, and distributed, as described above, multicomponent models separate various representations into discrete components. For example, the phonological loop stores phonological representations in a buffer separate from other representations (Baddeley and Hitch, 1974). Likewise, other researchers posit separate phonological and semantic buffers stemming from language mechanisms (Martin and Romani, 1994). These models are reminiscent of older, modular models of language comprehension and production that employ discrete stores and restricted interaction of information (Forster, 1985; Frazier, 1987). To fully capture the rich and interactive tapestry of language representations that are invoked in more current language research, multicomponent models would seem to require a combinatorial explosion of additional buffers for each form of interaction. In terms of parsimony and plausibility, this seems unlikely to be a tenable solution. Martin and Freedman (2001) offered a possible solution in which various language representations may interact in a multi-component memory model by passing the activity through layers with phonological and semantic buffers. This approach may allow more interaction but is also inconsistent with much language research, as it specifically implies that certain language representations are processed independently and in sequence (MacDonald and Seidenberg, 2006). As far as we are aware, no research has explicitly considered how different forms of interactive representations could be modeled in VWM in a manner consistent with language comprehension and production research. Even so, it is unclear how integrated representations and interactive processing could be implemented in a multicomponent account.

An important route for LTM effects on VWM performance in multicomponent models is redintegration, a process that rebuilds decaying memory traces from LTM (Roodenrys and Hinton, 2002; Roodenrys et al., 2002; Allen and Hulme, 2006; Clarkson et al., 2017). The redintegration mechanism not only rebuilds the phonological loop with phonological information from LTM (Clarkson et al., 2017), it also is the mechanism invoked to account for other LTM effects that go beyond phonological structure, including influences of word frequency and long-term knowledge of semantics and word co-occurrences on VWM (Hulme et al., 1997; Walker and Hulme, 1999; Roodenrys et al., 2002; Stuart and Hulme, 2009). In this view, the redintegration process must use LTM outside the phonological domain to shore up decaying phonological buffers. It is not clear how that process would work if LTM representations are highly integrated. Such a process would imply that phonological representations are first stripped from their richly integrated encoding in LTM, maintained in a separate phonological buffer, and then recombined with their integrated representations at the time of recall.

Currently, empirical evidence in favor of emergent (Postle, 2006; Acheson et al., 2011a; Buchsbaum and D’Esposito, 2019) and multicomponent accounts (for review, see Shallice and Papagno, 2019; Yue et al., 2019) has established little consensus. We recognize that many of the claims above are logical arguments, and further empirical evidence could prove some of our assumptions faulty. Proponents of emergent models should see language comprehension and production mechanisms as consistent with VWM systems that stem from a richly structured and integrated LTM (Acheson and MacDonald, 2009b; Jones and Macken, 2015; Hughes et al., 2016). Proponents of multicomponent models, however, may see these discussions of a rich language LTM and the processes that operate with it as simply more evidence for the sorts of information that could be encoded *via* language processes or that redintegration could use to reconstruct memory traces. Regardless, defining LTM representations is important for the advancement of memory models, and language models should provide insight into these LTM representations.

### Challenges for Limited Emergence

Perhaps one of the most persistent complaints against emergent accounts is their inability to handle aphasic patient data (Shallice and Papagno, 2019). Classically, patterns of behavior by patients with aphasia have been seen as evidence for the notion that STM and LTM are supported by distinct neural populations. Lesions to the medial temporal lobe have appeared to yield deficits of LTM with spared STM, typically assessed using lexical decision tasks and digit span tasks, respectively (Scoville and Milner, 1957; Penfield and Milner, 1958; Baddeley and Warrington, 1970; Warrington et al., 1971; Cave and Squire, 1992). In contrast, damage to left parietal regions have been interpreted to cause impairments in verbal recognition tasks and digit span tasks with spans greater than 1 or 2 while sparing other cognitive functions and LTM (e.g., Warrington and Shallice, 1969, 1972; Shallice and Warrington, 1970, 1974; Vallar and Baddeley, 1984). Thus, these studies of patients appeared to show a double dissociation of STM and LTM.

Some patient data may also support a dissociation between language processing and STM. For example, the patient K.F. reported in Warrington and Shallice (1969) exhibited strong repetition deficits with spared word knowledge, which would typically classify the patient as having conduction aphasia. However, given that the patient exhibited recognition deficits even when no verbal output was required by the task (i.e., pointing), Warrington and Shallice concluded that the patient’s impairment was not limited to language repetition. Later work reinforced this notion in patients with impaired phonological discrimination with spared word recognition and short sentence comprehension (Basso et al., 1982; Vallar and Baddeley, 1984; Silveri and Cappa, 2003) as well as in patients with dissociable speech and STM deficits (Martin and Breedin, 1992). In a similar way, more recent research has attempted to unconfound item and order memory (Attout et al., 2012; Majerus et al., 2015).

However, the putative pure deficits of STM are frequently tainted by subtle language impairments (Martin and Saffran, 1992). For example, Warrington et al. (1971) described a selective impairment of STM in a group of patients, yet those same patients exhibited difficulty in the repetition of abstract words, reading, and fluent speech. Vallar and Baddeley (1984) claimed to have found a pure deficit of STM in one patient, yet that same patient exhibited impaired comprehension of longer sentences compared to other participants. Even the patients identified with fluent speech also exhibited abnormalities. For example, the patient described by Shallice and Butterworth (1977) exhibited paraphasic errors in speaking names and had difficulty comprehending spoken discourse and written text. Furthermore, comprehension difficulty was exacerbated for complex sentences. Jacquemot et al. (2006) claimed to have found patients with a specific STM impairment, yet those same patients also exhibited difficulty in language comprehension tasks and sentence repetition tasks, resulting in phonological paraphasias. A truly pure deficit has proven quite elusive (though see Martin and Breedin, 1992). Rather than see these language deficits as stemming from a specific STM impairment, we see both as being driven by deficits in LTM. A complementary pattern is seen in other lines of research. For example, Hannula et al. (2006) found that hippocampal deficits cause impairments in relational processing at both short and long durations, upsetting prominent research suggesting that hippocampal activity is associated only with LTM. A strongly emergent perspective accords neatly with this data.

A recurrent theme in this review has been that the relationship between VWM and LTM depends on the nature of language LTM. Patient data is no exception. Reference to models of language production and comprehension reveals how apparent STM deficits could be captured by damage to LTM. Martin and Saffran (1992) presented the case of a patient with deep dysphasia who exhibited apparent errors of STM: difficulty producing nonwords and semantic errors in repetition. This patient exhibited fluent speech with semantic and phonological paraphasias. The researchers evaluated this patient’s performance through the lens of the Dell (1986) interactive spreading activation model of lexical retrieval. This model employs discrete representations of phonology, lexical entries, and semantics that interact in a bidirectional network. The model was able to produce human-like lexical selection behaviors. Critically, the model was able to capture putatively pure STM patient data solely through perturbation of the model parameters and without the inclusion of a distinct memory buffer. In this specific case, an increased decay rate reduced the ability of lexical representations to support lexical selection. The predictions afforded by this model were later confirmed in additional analyses of patient data by Martin et al. (1994; see also Dell et al., 1997), and patient recovery was also able to be modeled using the same framework (Martin et al., 1996). These results suggest that a specification of the LTM representations relevant to language comprehension and production may help test claims about the representational basis of VWM and its relationship to LTM.

Findings such as these point to the need for contact between theories of VWM and perspectives on long-term representations of serial order in language. That is, the extent to which the above or similar results affect VWM models depends on the hypothesized nature of LTM, particularly the extent to which LTM could contribute to representations of novel memoranda and their order. Language LTM captures relations between words and levels of linguistic representation and therefore allows generalization to new cases. Indeed, any linguistic input is novel in many ways, such as a new word-order, new speaker, new acoustic environment, and so on. By definition, the goal of language comprehension processes is to cope with novel input, and language production processes constantly generate novel utterances. The VWM literature offers a different perspective, with some claiming that buffers are needed explicitly to represent novel material (Norris, 2017). One challenge for memory research is the need to characterize a clear divide between “old” and “new,” especially given that novelty means very different things in different memory models. Distributed language models provide a key demonstration of the emergent perspective. In such models, novel stimuli are processed with respect to their similarity to prior experience, without any need for separate systems dedicated to handling the particularities of novel items or orders. In parallel, emergent models of VWM are capable of producing novel sequences just using LTM, without dedicated short-term buffers (e.g., Botvinick and Bylsma, 2005; Botvinick and Plaut, 2006, 2009). Perhaps greater adoption of graded representations of novelty could bridge the divide between language emergent and pure memory accounts. Important behavioral data linking graded phonotactic LTM to VWM (e.g., Tanida et al., 2015) and graded grammatical LTM to VWM (e.g., Jones and Farrell, 2018) already speaks to the usefulness of this approach.

## Implications for Language and VWM Research

We have cited a broad range of work in both VWM and in language comprehension and production, and one of the striking features of that work is how very little the fields have to say about each other. For example, it is completely uncontroversial that language comprehension and production processes are constrained by what is commonly called “verbal working memory capacity” in those fields, and yet the specific mechanisms posited in classic VWM models are, with only a few exceptions, absent from theorizing about how limited capacities shape language processes (for review and a different perspective, see Caplan and Waters, 2013). Similarly, while VWM accounts assume that VWM abilities must be used in everyday activities, the connection to actual theories of language use is equally scant. Here we discuss several fronts with more potential for interaction among the fields.

### Implications for Relating WM Assessments to Other Measures

The approach that we have advocated, in which performance on VWM tasks is heavily supported by language processes, which are themselves dependent on long-term knowledge, naturally leads to questions about what VWM tasks actually measure. This question is not only central to theories of working memory but also has enormous practical significance because there is wide usage of tasks that are described as VWM assessments in clinical and educational contexts—in typical and atypical child development, young adults, older adults, and patients with brain injury. Whereas some researchers have considered poor VWM performance as a cause of poor language skills, potentially ameliorated by working memory training (e.g., Ingvalson et al., 2015), our language-emergent VWM view suggests that poor VWM performance is a symptom associated with poor language skill. In other words, the abilities to encode, maintain, and order verbal information are skills that emerge from language use, and individuals who have higher language skills have richer LTM representations and more practiced comprehension and production processes (see also Jones et al., 2020). Thus, we can view tasks that are described as VWM tasks not as assessments of a separate VWM capacity but rather as measures of a person’s skill in encoding and maintaining verbal information. Consistent with this approach, there are now a number of reassessments of tasks that have previously been called “working memory tasks,” with arguments that they are better viewed as assessments of language skill, including but not limited to encoding, maintenance, and ordering. Tasks that have been reinterpreted in this way include reading span (MacDonald and Christiansen, 2002), digit span (Jones and Macken, 2015), nonword repetition (Edwards et al., 2004; Estes et al., 2007), sentence repetition (Klem et al., 2015), and immediate serial recall of word lists (Perham et al., 2009). In each of these examples, the argument has the same character. The apparent “verbal working memory task” does not measure a separate memory capacity but instead measures the quantity and quality of language skill and experience relevant to the specific demands of the task (see also Jones and Macken, 2018). Thus, nonword repetition performance can be traced to the knowledge of phonological patterns and vocabulary (Edwards et al., 2004; Gupta and Tisdale, 2009), digit span performance can be linked to prior experience with and statistical learning of digit sequences (Jones and Macken, 2015), and so on. The overarching conclusion from this work is that computational capacity to perform some task is not independent of long-term language knowledge and experience (MacDonald and Christiansen, 2002). That is an essential claim of anemergent perspective.

The emergent perspective also helps to elucidate so-called “brain training” research. If VWM is emergent from language LTM, then training VWM should only be beneficial (e.g., Soveri et al., 2017) if training improves relevant language skills. In contrast, VWM training should not be effective if it merely attempts to manipulate some independent notion of capacity. VWM training has been applied to therapeutic contexts, such as with aphasic patients, but the effectiveness of such interventions is unclear, driven in part by methodological limitations of single case studies (Zakariás et al., 2019). VWM treatments almost always employ linguistic stimuli of some sort, meaning they inherently provide some language practice. Therefore, VWM is rarely divorced from linguistic LTM in the training. VWM training research could benefit from a consideration of the emergent perspective defined hereby further developing language skills as opposed to separate memory capacity.

### Implications for Attention, Task Subcomponents, and Domain Generality

All theories of VWM have some mix of domain-specific and domain-general components. For example, the multicomponent model has the domain-specific phonological loop but also the general Central Executive, which guides behavior beyond the maintenance of phonological forms. Similarly, emergent views have domain-general attention and other cognitive control processes, but LTM can be domain-specific, in that linguistic knowledge need not have the same properties as a memory for smell or spatial relations. The specific emergent approach advocated here, in which language LTM and language comprehension and production processes underlie VWM functions, might initially seem strongly domain-specific in character, given the modular perspective that has pervaded language research. However, “emergent from language processes” need not be “domain-specific.” Indeed, there has been new interest in investigating how language use is supported by domain-general processes of attention and episodic memory (Nozari et al., 2016; Van de Cavey and Hartsuiker, 2016; Hepner and Nozari, 2019), and interest in how distinct brain networks must coordinate to accomplish language comprehension and other complex cognitive processes (Fedorenko et al., 2011; Fedorenko, 2014). Close ties with attention have long been a component of emergent models (e.g., Cowan, 1993), and researchers are now considering the interrelationships between language and attention mechanisms with respect to VWM (Majerus, 2019). More generally, there is real interest in considering the extent to which language production processes are related to or are themselves emergent from more general action planning processes or domain-general sequencing systems (Van de Cavey and Hartsuiker, 2016; Anderson and Dell, 2018; Guidali et al., 2019). Long-term ordering knowledge across domains (e.g., Kaiser, 2012; Van de Cavey and Hartsuiker, 2016) may inform sequence ordering, further tying together domain-general perspectives, emergent models, and language research. If language research continues to embrace more domain-general processes, this development could have substantial consequences for debates about the relationship between language processes and VWM, including distinctions between multicomponent and emergent accounts. That is, if VWM and language researchers both incorporate the same domain-general processes, then the distinction between multicomponent models and emergent models becomes less theoretically important.

Perhaps one of the most compelling examples of how domain-general processes affect language use and temporary maintenance may be seen in conversational turn-taking, which draws on episodic memory (Duff and Brown-Schmidt, 2012; Rubin et al., 2014) and cognitive control. Using data from recordings of conversations in 10 languages, Stivers et al. (2009) found that speakers typically begin speaking less than 500 ms after the previous speaker has ended their conversational turn. A number of researchers have argued that this closely time-locked behavior requires extensive attention, maintenance, and cognitive control because the next speaker simultaneously juggles a number of disparate tasks, some of which bear a close similarity to demands of VWM tasks. The conversational demands on the person who will soon speak include: comprehending the person currently speaking; planning a response and maintaining that utterance plan until time to speak; predicting the timing of the current speaker’s endpoint, which often involves predicting the actual words that the current speaker is likely to end on; and triggering an anticipatory in-breath and then exhalation to allow the speech to begin (de Ruiter et al., 2006; Torreira et al., 2015; Levinson, 2016). Not surprisingly, turn-taking and planning before speaking have high processing loads, as measured in a variety of methods (Kemper et al., 2011; Boiteau et al., 2014; Barthel and Sauppe, 2019). Thus, while a participant’s overall goals in a conversation and a VWM task are very different, it should be clear that the task demands of both activities overlap, including simultaneously encoding input while developing and maintaining plans to generate a response. Researchers are actively investigating the attention and cognitive control demands of language planning in advance of speaking, including serial ordering and monitoring of utterance plans (for review, see Nozari and Novick, 2017 and Fischer-Baum, 2018 for potential implications for VWM tasks). Some methods manipulating selective attention to individual words in a list could prove to be useful for new studies of both VWM tasks and more typical language production (e.g., Nozari and Dell, 2012; Nozari and Thompson-Schill, 2013). We see this research as complicating the domain-specific/general debates but also as an important arena for collaboration between VWM and language researchers.

### Implications for Language Production Research

The view that language production underlies maintenance of verbal information has significant implications for language production research. If every VWM study can be seen as a particular form of language production, the radically emergent perspective we describe has the potential to inform theories of language production. Interaction between the fields has long been evident at phonological levels. There has been keen interest in phonological level speech errors as important data for theories of serial ordering in language production (Dell, 1984; Dell et al., 1997), and there are extensive discussions of relationships between speech errors and recall errors in VWM tasks (Ellis, 1980; Hartley and Houghton, 1996; Page et al., 2007; Acheson and MacDonald, 2009a). In addition, VWM research has increasingly investigated the Hebb Repetition effect, the improved recall of repeated lists (Hebb, 1961; Oberauer et al., 2015). In parallel, production researchers have investigated the effects of learning on serial ordering and speech errors in production (Dell et al., 2000; Anderson et al., 2019). These investigations may be mutually informative, especially when placed in the context of computational models of ordering in VWM and models of language production which produce ordered sequences. As we have noted, some of these models have already suggested some parallels in ordering mechanisms between the two domains (Page and Norris, 2009; Hartley et al., 2016).

There are also potential parallels beyond the phonological level, relevant to questions concerning the relationship between words and their production in ordered sequences. MacDonald (2016) argued that of the three most obvious task demand differences between immediate serial recall and everyday language production (item list vs. coherent message, recall signal vs. spontaneous production, and producing exact list order vs. flexible language production), the latter was particularly important for understanding relationships between language production and VWM. Whereas serial recall, by definition, must be in the presented order, a hallmark of language production at the phrase or sentence level is serial order flexibility—that almost any message can be conveyed *via* several different words and word orders. This difference is informative when considering how interference among similar words can affect performance in language production and VWM tasks. Interference among list items leads to item omissions and re-ordering of list items in the recall; these are naturally treated as ordering errors, given the task demands in immediate serial recall (Baddeley, 1966; Page et al., 2007; though see Saint-Aubin and Poirier, 1999). Language production is also subject to interference among words, which leads to omissions and alternative word orders, compared to production conditions without interference (Gennari et al., 2012; Hsiao et al., 2014). These shifts and omissions are not considered errors but in some sense evidence of production skill, that is, evidence for how the speaker uses alternative ordering to maintain fluency in the face of interference. What is missing in this literature is a better understanding of interference during production planning and maintenance, and how alternative word orders emerge in the face of this interference. These questions seem ripe for insight from and collaboration with VWM research.

### Implications for Language Comprehension

Theories of language comprehension aim to explain how language percepts are recognized and interpreted. Important data in this endeavor have been measures of comprehension difficulty, or, more specifically, the relative difficulty of some kind of language compared to another. In the case of sentence-level comprehension research, the focus has been on why some kinds of sentences are harder than others, and VWM capacity has been a common explanatory factor in this field (MacDonald and Hsiao, 2018). Many researchers have invoked decay in VWM to explain comprehension difficulty of certain kinds of sentences, as the difficult sentences require integration over distant information that has degraded in working memory (Just and Carpenter, 1992; Gibson, 1998; Babyonyshev and Gibson, 1999; Grodner and Gibson, 2005). An alternative approach suggests that VWM and comprehension difficulty are constrained by interference rather than decay or capacity limitations (Lewis et al., 2006; Van Dyke and Johns, 2012; Glaser et al., 2013). This work emphasizes that both encoding and retrieval of information becomes more difficult with the increased semantic similarity between words, meaning sentences with more interfering elements are more difficult to comprehend (for review, see Van Dyke and Johns, 2012). This area is, therefore, another in which VWM research could inform comprehension, particularly the influence of decay and/or interference (Oberauer et al., 2016). More generally, though, while language comprehension researchers have often invoked VWM limitations in accounts of comprehension difficulty, they have not necessarily aligned themselves with particular VWM models of encoding, maintenance, and retrieval processes (for some exceptions, see Just and Carpenter, 1992; Martin and Romani, 1994; Lewis et al., 2006; Caplan and Waters, 2013).

At least initially, very few accounts of language comprehension ascribed a major role for experience in language comprehension difficulty. These accounts were, at least in principle, aligned with a multi-component perspective. A separate, temporary store, separate from long-term language knowledge, provided a bottleneck in encoding and maintenance that could explain comprehension difficulty. More recently, a number of researchers have suggested that both VWM capacity and language experience are important components in processing difficulty (Demberg and Keller, 2008; Staub, 2010). In a more fully emergent approach of VWM, the capacity to encode and maintain information (whether for everyday language use or a working memory task) is not independent of long-term memory, and thus not independent of experience with language (McClelland and Elman, 1986; MacDonald and Christiansen, 2002; Botvinick and Plaut, 2006; Acheson and MacDonald, 2009a; Jones and Macken, 2015). We see this emphasis on experience-based capacity as a basis for investigating parallels between comprehension processes and VWM. Moreover, the emphasis on experience also casts language use and memory as intertwined, learned skills, as noted in the discussion of revised interpretations of VWM tasks above. For example, memory researchers have noted relationships between novel word learning and the Hebb repetition effect (Szmalec et al., 2009). If word representations are highly intertwined, as our emergent perspective claims, then sensitivity to the Hebb repetition effect and novel word learning should exhibit exploitation of statistical regularities between different sources of information (e.g., Cassidy and Kelly, 1991; Nygaard et al., 2009) rather than mere memory capacity of the learner.

## Conclusions

In this article, we have aimed to describe the rich nature of linguistic LTM and its consequences for VWM. While Ebbinghaus (1885) had inklings that LTM could not be fully set aside in studying VWM, we have suggested that the linkage between language LTM and VWM is far stronger than he imagined, in part because LTM has a different quality than he and many others had hypothesized. A more thorough understanding of the nature of language processing, attention, and LTM, we claim, will accelerate the advancement of both VWM and language research. We have argued that words are not unrelated islands in LTM representations, and therefore they should not be treated as isolated items in VWM research. We have further argued that the processes of language comprehension and production underlie a person’s ability to encode, maintain, and order verbal information. These skills are essential for everyday language use, change with experience and the richness of LTM, and are brought to bear on VWM tasks. On this view, VWM and language research should be mutually informative.

## Author Contributions

SS and MM contributed to all phases of writing this review.

## Conflict of Interest

The authors declare that the research was conducted in the absence of any commercial or financial relationships that could be construed as a potential conflict of interest.
